# Neuroprotection in Acute Ischemic Stroke: A Battle Against the Biology of Nature

**DOI:** 10.3389/fneur.2022.870141

**Published:** 2022-05-31

**Authors:** Sherief Ghozy, Abdullah Reda, Joseph Varney, Ahmed Sallam Elhawary, Jaffer Shah, Kimberly Murry, Mohamed Gomaa Sobeeh, Sandeep S. Nayak, Ahmed Y. Azzam, Waleed Brinjikji, Ramanathan Kadirvel, David F. Kallmes

**Affiliations:** ^1^Department of Neuroradiology, Mayo Clinic, Rochester, MN, United States; ^2^Nuffield Department of Primary Care Health Sciences and Department for Continuing Education (EBHC Program), Oxford University, Oxford, United Kingdom; ^3^Faculty of Medicine, Al-Azhar University, Cairo, Egypt; ^4^School of Medicine, American University of the Caribbean, Philipsburg, Sint Maarten; ^5^Qena Faculty of Medicine, South Valley University, Qena, Egypt; ^6^Medical Research Center, Kateb University, Kabul, Afghanistan; ^7^Barry University, Miami, FL, United States; ^8^Faculty of Physical Therapy, Sinai University, Cairo, Egypt; ^9^Faculty of Physical Therapy, Cairo University, Giza, Egypt; ^10^Department of Internal Medicine, NYC Health + Hospitals/Metropolitan, New York, NY, United States; ^11^Faculty of Medicine, October 6 University, Giza, Egypt; ^12^Department of Neurosurgery, Mayo Clinic Rochester, Rochester, MN, United States

**Keywords:** acute ischemic stroke, neuroprotection, ischemia, therapy, management

## Abstract

Stroke is the second most common cause of global death following coronary artery disease. Time is crucial in managing stroke to reduce the rapidly progressing insult of the ischemic penumbra and the serious neurologic deficits that might follow it. Strokes are mainly either hemorrhagic or ischemic, with ischemic being the most common of all types of strokes. Thrombolytic therapy with recombinant tissue plasminogen activator and endovascular thrombectomy are the main types of management of acute ischemic stroke (AIS). In addition, there is a vital need for neuroprotection in the setting of AIS. Neuroprotective agents are important to investigate as they may reduce mortality, lessen disability, and improve quality of life after AIS. In our review, we will discuss the main types of management and the different modalities of neuroprotection, their mechanisms of action, and evidence of their effectiveness after ischemic stroke.

## Introduction

The rapid development of neurologically related deficits is a potent indicator of acute ischemic stroke (AIS) (1). This happens as a result of the presence of an acute vascular etiology that lasts for at least 24 h and focally affects the central nervous system, inducing major disturbances to the related functions of the affected areas that may even end with mortality (1, 2). In general, stroke is the second most common cause of global death following coronary artery disease, and estimates show an annual incidence of 12.2 million (3). In addition, estimates show that it affects nearly 795,000 patients in the US (4). Reports also showed that stroke is the third most common contributor to disability and increased morbidities in approximately half of the stroke survivors over 65 years of age (4, 5).

Time is crucial in managing stroke to reduce the rapidly progressing insult of the ischemic penumbra and the severe neurologic deficits that might follow (6, 7). Hence, early management provides a window of opportunity for protecting against the development of severe complications and death, subsequently enhancing the prognosis (8). Furthermore, evidence concerning the management of stroke is changing quickly. Many studies are being published that report novel technical modalities and discoveries that can help improve the outcomes of stroke (9, 10). AIS has many management routes, including intravenous (IV) thrombolytic drugs such as recombinant tissue plasminogen activator (rtPA), control of body temperature and blood sugar, and blood pressure reduction of intracranial tension and neuroprotective agents (11). While IV thrombolytic agents help restore blood flow through an occluded vessel, neuroprotective agents are very important for maintaining the function of neurons surrounding dead brain tissue and, hence, limit after-stroke deficits (12).

There is rising interest in knowing more about neuroprotective agents, as shown in the currently ongoing studies listed in Table 1. These studies aim to investigate neuroprotective agents that can be beneficial to intervene against ischemic stroke and are the intended primary outcomes of these studies. In addition, data about neuroprotection approaches can now be easily accessed through multiple national and international registries that can help researchers globally. However, there is still no consensus about the unified management modalities to achieve the best prognosis. Currently, the US Food and Drug Administration (FDA) has not approved a specific treatment modality as neuroprotective therapy for ischemic stroke. This study aims to elaborate on the current gold standard management modalities of AIS. We will also discuss the latest reported evidence about the neuroprotective modalities as per evidence from studies in the literature.

**Table 1:** List of most prominent ongoing clinical trials.

**Registration number**	**Title of the study**	**Type of study**	**Type of neuroprotection**	**Primary outcome**	**Results**
NCT04061577	Transcranial Direct Current Stimulation as a Neuroprotection in Acute Stroke Before and After Thrombectomy (TESSERACT-BA)	RCT	Direct current stimulation	Primary safety outcome- rate of symptomatic intracranial hemorrhage	Ongoing
NCT03481777	Remote ischemic conditioning in patients with acute stroke (RESIST)	RCT	RIC	Clinical outcome (mRS) at 3 months in acute stroke patients	Ongoing
NCT02767778	Low-frequency Pulsed Electromagnetic Fields (ELF-MF) as Treatment for Acute Ischemic Stroke (I-NIC)	RCT	Low frequency pulsed electromagnetic fields	Change in the volume of the ischemic lesion measured by MRI	Ongoing
NCT04266639	Rheo-erythrocrine dysfunction as a biomarker for RIC treatment in acute ischemic stroke (ENOS)	RCT	Rheo-erythrocrine dysfunction as a biomarker for RIC treatment	Red blood cell deformability will serve as a biomarker of the conditioning response and predictor of the clinical outcome in stroke patients	Ongoing
NCT04554797	Regional hypothermia in combination with endovascular thrombectomy in acute ischemic stroke (RE-HIBER)	RCT	Regional hypothermia	Rate of any major adverse events	Ongoing
NCT03375762	REMOTE ischemic perconditioning among acute ischemic stroke patients (REMOTE-CAT)	RCT	Remote ischemic preconditioning	Dependency based on mRS through first 3 months	Ongoing
NCT03347786	Verapamil for neuroprotection in stroke	Single group assignment	Verapamil	Serious adverse event	Ongoing
NCT03915431	A Study of NCS-01 in patients with acute ischemic stroke	RCT	NCS-01 infusion	Number of participants with adverse events -Safety by Incidence of Treatment-Emergent Adverse Events	Ongoing
NCT03876119	Intraarterial alteplase vs. placebo after mechanical thrombectomy (CHOICE)	RCT	Intra-arterial alteplase	The primary outcome will be the proportion of patients with a mRS 0 to 1 at 90 days	Ongoing
NCT02453373	Helping stroke patients with thermosuit cooling (SISCO)	Non-randomized clinical trial	Rapid induction of therapeutic hypothermia (32-34°C) using the Life Recovery Systems ThermoSuit System Magnesium sulfate, intravenous administration	Feasibility of cooling as indicated by percentage of patients cooled to target within 1 h of start of cooling. Neurological outcome as indicated by NIHSS Safety of the cooling treatment as indicated by rates of significant adverse events Neurological outcome as indicated by mRS score Change in neurological outcome as indicated by NIHSS Change in neurological status as indicated by mRS	Ongoing
NCT03804060	REperfusion with cooling in cerebral acute ischemia II (RECCLAIM-II)	RCT	Cooling with the ZOLL Circulation Proteus catheter and the ZOLL Intravascular Temperature Management system to initiate and maintain hypothermia for 6 h as an adjunct to endovascular recanalization	Percentage of test arm patients achieving target temperature Mean door-to-recanalization time Rate of hemorrhagic conversion in each arm within 36 h of recanalization	Ongoing
NCT03284463	Safety and efficacy of glibenclamide combined with Rt-PA in acute cerebral embolism (SE-GRACE)	RCT	Glibenclamide is administered with a loading dose of 1.25 mg within 10 h of stroke onset, orally or through gastric tube, followed by 0.625 mg every 8 h for 5 days	Functional outcome: The proportion of mRS of 0 to 2 points	Ongoing
NCT02864953	Phase 3 Study to evaluate the efficacy and safety of intravenous BIIB093 (Glibenclamide) for severe cerebral edema following large hemispheric infarction (CHARM)	RCT	Intravenous glibenclamide	Percentage of participants with improvement in functional outcome at day 90 Assessed via the mRS	Ongoing
NCT03753555	The effect of intensive statin in ischemic stroke with intracranial atherosclerotic plaques (INSIST-HRMRI)	RCT	Atorvastatin Calcium 20 mg every day for 12 months in the comparator group. Atorvastatin Calcium 40–80 mg every day for 6 months	Changes in remodeling index after the statin treatment Changes in plaque burden after the statin treatment Changes plaque composition in after the statin treatment	Ongoing
NCT04275180	Clinical study of argatroban in the treatment of acute progressive ischemic stroke	RCT	Argatroban plus standard medical treatment	Proportion of patients with a 3-month mRS score ≤ 3	Ongoing
NCT04425590	The benefit of add on DLBS1033 for ischemic stroke patient	RCT	DLBS 1033 (disolf) 490 mg tablet 3 times daily	Improvement in mRS scores at hospital discharge Improvement in mRS scores at 30 days Improvement in NIHSS scores at hospital discharge Improvement in NIHSS scores at 30 days Improvement in BI scores at hospital discharge Improvement in BI scores at 30 days	Ongoing
NCT03724110	Telestroke for comprehensive transient ischemic attack care in acute stroke ready hospitals (TELECAST-TIA)	Cohort study	Telestroke is an audiovisual communication network that allows for coordination of stroke care from a distant 'hub' site (the telestroke provider location) to an originating 'spoke' site (patient location)	Composite score of TIA treatment (%)	Ongoing
NCT03868007	Protective effects of RIC in elderly with acute ischemic stroke complicating acute coronary syndrome (RIC-ACS)	RCT	The RIC procedure during which bilateral arm cuffs are inflated to a pressure of 200 mm Hg for 5 cycles of 5 min followed by 5 min of relaxation of the cuffs	Death rate	Ongoing
NCT03993236	Study on rosuvastatin + ezetimibe and rosuvastatin for LDL-C goal in patients with recent ischemic stroke	RCT	The experimental group is orally administered with rosuvastatin 10 mg plus ezetimibe 10 mg combination once daily for 90 days	The percentage of patients with LDL-C decreased more than 50% at 90 days (±14 days) compared to baseline	Ongoing
NCT03287076	Trial of EXenatide in Acute Ischaemic Stroke (TEXAIS)	RCT	Patients will receive exenatide injections	Improved neurologic outcome	Ongoing
NCT03635177	Effect of remote ischemic conditioning on vascular health in stroke patients (VISP)	RCT	The participant will conduct remote ischemic preconditioning by use of an automated cuff device placed on the upper arm which inflates to 200 mm Hg and occludes blood flow The procedure is conducted on one arm 4 x 5 min per day Each 5 min occlusion period is interspersed by 5 min	Assessment of brachial artery dilation after 5 min occlusion of upper arm by cuff	Ongoing

*BI, Barthel index; LDL-C, low-density lipoprotein cholesterol; MRI, magnetic resonance imaging; mRS, modified Rankin Scale; NIHSS, National Institutes of Health Stroke Scale; RCT, randomized controlled trial; RIC, remote ischemic conditioning; rtPA, recombinant tissue plasminogen activator; TIA, transient ischemic attack*.

## Methods

The current literature review was conducted based on a comprehensive search strategy through various electronic databases, including EMBASE, Medline, and Cochrane library. No restrictions regarding the year of publication or country of the population were applied. However, we only included studies that were published in English. The search strategy was built on different relevant keywords obtained from relevant investigations and using the medical subject headings (MeSH). The formulated search strategy was modified based on the terms and conditions of each database to obtain the most relevant number of investigations. Finally, we conducted an additional manual search within the references of relevant articles and reviews to obtain any missed articles during the search strategy. We intended to include studies that reported neuroprotective agents that can be used for AIS patients.

### Consequences of Blood Supply Disruption

After an ischemic stroke has occurred, various pathologic mechanisms occur, ultimately leading to brain tissue damage. Knowing how ischemia affects the brain is critical in assessing how to treat patients suffering from ischemic stroke. Once disruption of the blood supply has occurred, the cells of the brain die by several mechanisms. First, the diminished blood flow of the infarcted tissue leads to necrosis in adjacent penumbra tissue and decreased cellular metabolism (13). Second, when energy levels are depleted through the decreased generation of adenosine 5′-triphosphate, leading to sodium/potassium pump failure, the depolarization of the cell allows for calcium influx, resulting in cell apoptosis *via* the intrinsic pathway (14).

Evidence indicates that different factors that play a role in the level of ischemic brain tissue damage are neuronal excitotoxicity, peri-infarct depolarizations, local inflammation, and the accumulation of oxidative stress (15, 16). In this context, oxygen deprivation and blood flow loss lead to a near-immediate halt on neuroelectric activity and a loss of ion homeostasis (17–19). This ultimately lowers the energy stores by lowering adenosine triphosphate production (*via* mitochondrial dysfunction), leading to failure in the sodium-potassium pump. The cell's ion gradient is then compromised, leading to increased release of glutamate and aspartate at toxic concentrations (17–19). The increased glutamate release causes the activation of ionotropic receptors, leading to calcium influx and overload. This eventually leads to cellular damage, neuronal over-excitement, and ultimately cell death (20, 21).

Besides, free radical oxygen species (ROS) and calcium play a role in increasing levels of inflammatory cytokines, chemokines, and endothelial cell adhesion molecules, which assist in inflammatory cell migration and the upregulation of specific proinflammatory genes (15, 22, 23). When the sodium/potassium pump is impaired, as are glutamate and glucose transporters when free radicals are formed, mechanisms behind this are believed to be due to 4-hydroxynonenal production, lipid peroxidation, and their role in inducing apoptosis (24).

**Figure 1:**
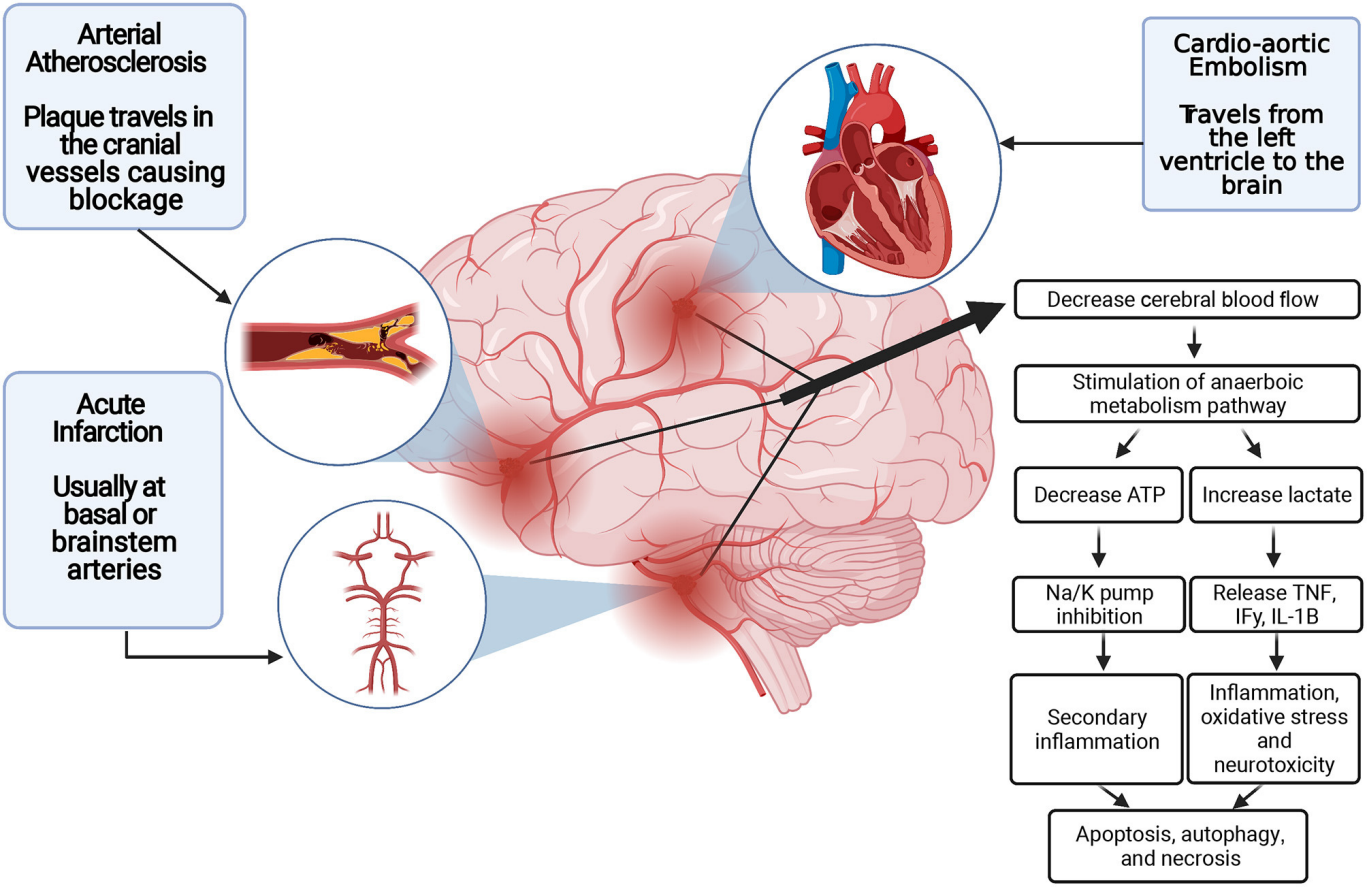
Blood supply disruption and ischemic stroke pathogenesis. ATP indicates adenosine triphosphatase; IFγ, Interferon gamma; IL-1B, interleukin 1B; K, potassium; Na, sodium; TNFα, tumor necrosis factor α.

Another factor linked to brain cell death and a decreased patient prognosis following an ischemic stroke is increased cerebral edema (25). With the brain being in such a confined space, postinflammatory edema can lead to rapid brain tissue damage. In addition, various antiport channels have been shown to lead to cerebral edema after ischemic stroke. These channels include the sulfonylurea receptor 1–transient receptor potential melastatin 4 (SUR1-TRPM4) cation channel and the aquaporin-4 channel (26). Thus, the limitations of post-ischemic stroke edema could play a neuroprotective role (Figure 1).

### Gold Standards for the Management of AIS

#### Recombinant Tissue Plasminogen Activator

The American Heart Association/American Stroke Association (AHA/ASA) guidelines have emphasized the efficacy of rtPA in the early management of AIS and a notable improvement in outcomes. rtPA helps recanalize the occluded blood vessels through the lysis of the blood clot and subsequently the reperfusion of brain tissue. The best results of rtPA can be obtained when administered intravenously within 3 h from the onset of symptoms of AIS (27, 28). In another study, a longer time window of 4.5 h was shown to be effective and safe in the management of AIS (29). In addition, several clinical trials (e.g., NINDS, ECASS, ECASS II, ATLANTIS, PROACT, and others) have studied the safety and effectiveness of using rtPA with a time window of 6 h (30, 31). However, some adverse effects were reported from the administration of rtPA in a 6-h time window. The most severe adverse effects included increased mortality and a high incidence of intracranial hemorrhage, which occurred less frequently in the 3-h or 4.5-h time windows (32). Also, rtPA is contraindicated in the presence of intracranial hemorrhage (33). Approximately 3 to 8% of patients with an ischemic stroke are eligible for IV thrombolytic therapy, with only 20 to 66% achieving complete recanalization. Furthermore, over 30% of patients who did achieve complete recanalization suffer from a neurologic injury due to re-occlusion (34, 35).

More recent clinical trials emphasized the importance of imaging-guided thrombolysis in patients with AIS, with a non-definite time of onset. Although this approach will enhance the efficacy of detecting thrombolysis candidates (36), some limitations, including far distance to the scanner, time spent in screening, and cost, might reduce this efficacy. Moreover, the eligibility for IV-tPA was evaluated by CT-P imaging in the Thrombolysis Guided by Perfusion Imaging up to 9 H after Onset of Stroke (EXTEND) clinical trial. The authors demonstrated that this approach could extend revascularization time to 24 h and the safety and efficacy of IV-tPA to 9 h (37).

More recent trials also investigated using a novel thrombolytic agent, tenecteplase, with high fibrinogen specificity, increasing its efficacy. Moreover, it has a long half-life and can be administered as a single bolus. This has been indicated in the Tenecteplase vs. Alteplase before Thrombectomy for Ischemic Stroke (EXTENT-IA-TNK) trial, indicating better functional outcomes and higher reperfusion rates than patients with AIS that were administered alteplase and were eligible for endovascular thrombectomy (EVT) (38, 39). Other studies also concluded that the efficacy and safety of tenecteplase and alteplase are similar when given to patients without large vessel occlusion (LVO) (40, 41). However, this modality is not FDA-approved and is not recommended by the AHA/ASA guidelines at the same level as alteplase.

#### Endovascular Thrombectomy

Treatment of ischemic stroke caused by LVO has been routinely managed using endovascular approaches (42–46). This approach consists of the interventionalist guiding a microcatheter from the groin or arm into the blood clot under X-ray guidance. However, as with thrombolytic pharmacologic therapy, many people are deemed ineligible for endovascular treatment. A study found that only 7 to 13% of patients with ischemic stroke were eligible candidates, with poor outcomes reported in up to 49% of participants and failure seen in 41% (42–47). Ineligibility is based on the timeframe, with large and small occlusions having a 6- and 24-h cutoff, respectively (48, 49). Another study showed the prior usage of rtPA before EVT has not shown substantial benefit in the outcomes of this procedure (50). Notably, the product of an EVT is not readily available due to its highly specialized nature with specific imaging equipment required to see cranial vessels (51).

Strategies to rescue ischemic stroke now include transduction blockage in downstream pathways. This includes the binding of glutamate to inhibit receptor stimulation and the antagonism of detrimental *N*-methyl-D-aspartate (NMDA) receptors selectively. These various mechanisms have all shown promise for ischemic stroke patients with minimal adverse effects (52).

### Preclinical Models for Studying Neuroprotection

Neuroprotection research has found that testing works in animal models, but the outcomes in clinical trials have been disappointing. A rat stroke model (middle cerebral artery occlusion) revealed that even single whisker stimulation could induce complete protection of the rat cortex (53). Over 1,000 neuroprotective agents have been tested in preclinical stroke studies, with many showing promising success (54). Nearly 200 neuroprotective clinical trials have been performed, but only a tiny percentage of them have been successful (16, 54, 55). Sadly, substantial gaps in the literature still exist between animal studies and clinical trials on stroke. The aspects they can test help us understand the physiologic parameters of new drugs while giving our neuroprotective targets validity (56), although much is still lacking. For instance, it is known that the rodent most commonly used in preclinical stroke research cannot mimic the typical AIS patient (57).

Furthermore, some facets of ischemic stroke are not feasible in animal models, including preventative strategies (56). Although comorbidities such as diabetes and hypertension can be induced into the rodents being tested, the cost is high (58). Thus, most mice being tested are young and healthy males, although female mice account for a majority of stroke mortalities (59). These preclinical stroke model differences have led to a large gap between animal studies and clinical trials (56). With the utility of comorbidities and preclinical randomized control trials (RCTs) and studying the clinical setting, we may be able to close the gap leading to an increased understanding of AIS (56).

### Neuroprotective Strategies and Agents

The main aim of the management of AIS is to restore the blood flow to the affected ischemic part of the brain. In contrast, the secondary goals include the modulation of any process that may aggravate the irreversible nerve cell damage induced by ischemia to the surrounding ischemic area and enhance the functional outcomes of the disease (56). Neuroprotection is studied based on understanding the pathophysiology of AIS to achieve these goals. Neuroprotection is the process that prevents the cellular injury of the brain in the setting of ischemia. This protection encompasses either interventional or pharmaceutical treatments (16). As a result, any pharmacologic or interventional technique that mitigates or prevents the harmful molecular and cellular activities that contribute to permanent cerebral ischemia is referred to as *neuroprotection* (16). Neuroprotective and collateral treatment in stroke tends to be more successful when delivered as soon as possible after the onset of symptoms. To minimize the time from stroke to needle, care should preferably begin before admission to the hospital (60). American Heart Association guidelines currently do not promote pharmacologic agents with putative neuroprotective actions, except for research purposes (33). Having adequate collateral blood flow to the brain has been shown to decrease infarct expansion leading to improved patient outcomes and prognosis in ischemic stroke patients (61–63). Thus, utilizing collateral blood flow to the ischemic area of the brain also presents physicians with another treatment option other than immediate clot thrombosis and recanalization (64).

Furthermore, studies show the importance of neuroprotection therapy together with reperfusion modalities. In this context, a recent systematic review showed that 5 out of 15 clinical trials indicated that patients treated with neuroprotection and reperfusion therapy had good clinical outcomes (65). These findings indicate the promising clinical future of using neuroprotection therapy for patients with AIS.

#### Therapeutic Hypothermia

Therapeutic hypothermia has proved its efficacy as a neuroprotective strategy with promising outcomes in protecting nerve injuries in humans. Therapeutic hypothermia as a neuroprotective strategy was shown to improve the prognosis of neurologic injuries in experimental animals and cardiac arrest patients (66). It works by many mechanisms to achieve neuroprotection. Reducing the basal metabolic rate through preserving glucose, PH, and ATP in the brain tissue is a critical mechanism for establishing therapeutic hypothermia's role as a neuroprotectant (67, 68). Other mechanisms include the inhibition of free radicals and nitric oxide generation (both play a role in nerve cell damage), modulation of the inflammatory processes that accompany and may aggravate the neuronal injuries, in addition to inhibition of nerve cell apoptosis and the protection against excitotoxic agents (as reported in some rat model studies) (69–71). Therapeutic hypothermia can be achieved through two different methods, either physical hypothermia or pharmacologic (drug-induced) hypothermia. Physical hypothermia can be obtained locally through a cooling helmet to get local and selective brain hypothermia (72) or generally through cooling blankets or ice pads and rapid infusion of cold saline (73).

On the other hand, many classes of drugs, such as diazepam, dopamine agonists, opioids, adenosine derivatives, and others, were reported to induce hypothermia (74, 75). However, therapeutic hypothermia has proved its efficacy as a neuroprotective treatment; it was found that therapeutic hypothermia in combination with other neuroprotectants with various mechanisms was more successful and gave better outcomes than administration of a single neuroprotective agent (16, 76). So it is advisable to apply therapeutic hypothermia in combination with other agents (77).

#### Ischemic Conditioning

Triggering mechanisms implicated in endogenous ischemic tolerance represents a very promising strategy to protect neurons against an ischemic insult (78). In this context, preconditioning, or the more clinically feasible postconditioning, with a brief period of ischemia or with a chemical challenge, is a practical approach to decrease neuronal death caused by a more severe ischemic episode (79, 80). These studies have found that neuronal cell death is minimized through preconditioning or postconditioning of a short period of ischemia or chemical challenge (79, 80). Meaning that once there has been a mild neurotransmitter stress (i.e., glutamate), the patient is left in a state tolerant of a more severe level of glutamate exposure.

Previous *in vitro* studies have established that preconditioning induced by glutamate or sublethal oxygen-glucose deprivation in cultured neurons is mediated by activation of NMDA receptors (81–83). It was also discovered that ischemic preconditioning increased γ-aminobutyric acid (GABA) production and release during lethal cerebral ischemia and its effects on glutamate neurotransmission (78, 84). Ischemia tolerance relies on conformational changes of both the presynaptic and postsynaptic GABA receptors causing a shift in the glutamate and GABA neurotransmitter balance (85). Importantly, different sodium-calcium exchanger isoforms have also been found to play a role in both precondtioning and postconditioning (86).

#### 3K3A-Activated Protein C

Activated protein C (APC) is an anti-inflammatory and anticoagulant factor that was reported to have a neuroprotective role in ischemic stroke in many animal studies and a few human studies. It conducts its neuroprotection by inhibiting cell apoptosis through its action on endothelial protein C receptor and protease-activated receptor-1 (PAR-1) (87). The PAR-1 agonist (3K3A-APC) has shown promise in previous clinical trials in lowering neurologic injury and promoting vascular integrity. One study found that when 3K3A-APC was used in combination with rtPA, hemorrhage occurrence was reduced (88). It was also found that infarct size and an increased therapeutic window were seen when utilizing 3K3A-APC with rtPA in older female rodents suffering from acute high blood pressure. However, no additional benefit was seen if rtPA was not administered within the first 4 h after the onset of ischemia. More recent trials did appear to show the same lowered hemorrhage rates, although the authors stated experimental confirmation should be completed (89). However, human studies about 3K3A-APC brain cytoprotection in ischemic stroke patients are still deficient and need more evaluation and assessment before using this agent in humans safely. Hence, many efforts are directed to examine the effectiveness of the signaling-selective 3K3A-APC variant to upgrade its use from the preclinical to the clinical stage (90–92).

#### NXY-059

NXY-059 is an agent that reduces free radicals. Free radicals are involved in brain cell death in AIS (15, 22–24). Through its free radicals trapping property, NXY-059 was reported in a clinical trial (SAINT I) to have substantial neuroprotective efficacy when administered intravenously within 6 h after stroke onset, reducing the rate of 90-day disability (93). Contrastingly, the SAINT II clinical trial showed a 72-h IV infusion of NXY-059 in patients with acute stroke which was shown to be ineffective in improving stroke outcomes (94). After the confusing and controversial results from SAINT I and SAINT II, Antonic et al.'s (95) recent experiments about the efficacy of NXY-059 to reach a clear conclusion about its effectiveness have reported the failure of NXY-059 as a neuroprotective agent.

#### Nitric Oxide

Vascular nitric oxide concentrations are low in acute stroke and associated with poor outcomes, therefore, allowing the possibility that supplemental nitric oxide might be beneficial (96). Preclinical stroke studies found nitric oxide donors improved regional cerebral blood flow, and the lesion size was reduced if administered rapidly (22, 97). Therefore, a nitric oxide donor transdermal glyceryl trinitrate has been tried in the setting of acute stroke and randomized trials have shown that it was effective in improving multiple acute stroke hemodynamic parameters. These positive results included decreased central and peripheral blood pressure, decreased pulse pressure, and decreased blood pressure at 24 h while improving the augmentation index (96, 98–101). The timeframe of administration of transdermal glyceryl trinitrate has been shown to vary between different test studies, with some suggesting its utility within 4 h of stroke (101), while others say within 6 h (98, 102). A large study in the United Kingdom showed that the utility of prehospital transdermal glyceryl trinitrate in the prehospital setting did not improve patient outcomes with assumed stroke (103).

#### Magnesium Sulfate

Magnesium sulfate was documented to be a potent neuroprotectant, especially if given antenatally, where it considerably reduced the incidence of cerebral palsy and death (104). Since that study, many preclinical and clinical studies investigated its neuroprotection in ischemic stroke (105). Magnesium sulfate's neuroprotective effects come mainly from its antagonism to NMDA glutamate receptors, enhancing the blood flow to the ischemic areas and improving the metabolic recovery after ischemia (106–108). Many RCTs targeted the safety and probability of magnesium sulfate as one type of management in ischemic stroke (109–112). For example, the recent FAST-MAG trial showed the safety of prehospital application of magnesium sulfate therapy within 2 h from stroke onset. But it also revealed a slight improvement in stroke outcomes in the 90-day disability measured by scores on the modified Rankin scale (112).

#### NA-1 Delivery

When glutamate levels are elevated in the extracellular space, it leads to the activation of kainate glutamate, NMDA, and α-amino-3-hydroxy-5-methyl-4-isoxazole propionate acid receptors. This results in the influx of calcium which ultimately leads to damage of cells in ischemic infarct (113). Thus, blocking these receptors has been theorized as a possible treatment for ischemic stroke. However, the utility of the NMDA receptor antagonists on ischemic stroke has been shown to have a very tight timeframe for inducing positive effects. The receptors are significantly impaired before 2 h of ischemia onset, while reduced NMDA receptor density is observed in periods exceeding 2 h (114). Thus, the primary reason for failure in the clinical trials is due to immediate toxicity rather than the delayed recovery of the NMDA antagonist blockage mechanism (115).

The drug NA-1 has been shown to lessen the excitotoxic signaling of NMDA postsynaptic signaling. This is accomplished through targeting the 95 protein (116). Intervention studies before endovascular aneurysm surgery have shown that pretreatment of NA-1 before an operation decreased the frequency of iatrogenic stroke (ENACT trial) (117). This drug has also been tested in macaques known to have genetics similar to humans with positive behavioral findings and magnetic resonance imaging post-ischemic stroke (118). NMDA receptor stimulation, rather than inhibition, was influential in the subacute period following stroke, leading to plasticity and memory formation (119, 120). Despite the blockade of mechanisms of post-ischemic repair, the primary reason for the failure of clinical trials with NMDA antagonists was related to immediate toxicity rather than delayed recovery. The latest RCTs of EVTs performed in 2015 recognized critical points in the clinical trial design (faster revascularization and selection of patients with a limited core or presence of occlusion) and yielded promising results (42–46).

#### Statins

Statin therapy has been shown to have utility in both primary and secondary stroke prevention (121, 122). Furthermore, statins have also improved recovery times and survival 1-week post-AIS (123). However, after the onset of stroke, the discontinuation of statin therapy has been shown to worsen outcomes, whereas the initiation of statins has been shown to do the opposite (124). In NeuSTART, the administration of lovastatin (8 mg/kg) 3 days post-AIS was well tolerated (125). In the multicenter ASSORT RCT performed in Japan, Yoshimura et al. compared early (within 24 h) or delayed (on day 7 of AIS onset) administration of statin therapy in patients with AIS and dyslipidemia. The RCT had shown an insignificant difference and almost the same outcome in both early and delayed administration of statins (126).

#### Glyburide

Glyburide is a second-generation sulfonylurea used as an oral hypoglycemic drug in control of diabetes. It has been demonstrated that glyburide effectively blocks the SUR1-TRPM4 non-selective cation channels (127, 128). These channels are upregulated and reported to mediate cytotoxic brain swelling and subsequently brain cell oncotic necrosis in the setting of ischemia (129). Therefore, the blockade of these receptors can assist in nerve cell protection in cases of AIS. Results of preclinical animal studies emphasize the theory of glyburide's role as a neuroprotectant in AIS (127, 130). GAMES-RP, a double-blind, randomized phase II trial, reported the safety and well-tolerated use of glyburide in critically ill patients without substantial improvement of the disability or the need of craniotomy to alleviate brain edema compared to placebo (131, 132). An exploratory analysis of the GAMES-RP trial followed and showed the influential role of IV glyburide therapy in improving the survival rates in patients 70 years or younger with acute hemiplegic infarction (133). Further investigation in a phase III trial is warranted.

#### Insulin

Hyperglycemia is common when the infarct size is large in AIS patients (134). Conversely, persistent hyperglycemia following stroke was found to exacerbate the infarct size and subsequently give poor functional outcomes (133, 135). Additionally, Bruno et al. (136) reported in the NINDS rt-PA Stroke Trial that the presence of hyperglycemia in patients with stroke was associated with an increased risk of hemorrhagic events regardless of rtPA therapy. High blood glucose was also found to negatively impact the mortality rates in events of AIS (137). Hence, controlling blood glucose levels in stroke patients is crucial in improving the outcomes and protecting nerve cells from damage after exposure to ischemia. Insulin administration in cases of AIS was found to have beneficial neuroprotective effects. Reversal of the pathology produced by hyperglycemia [e.g., reducing the blood flow to the ischemic penumbra and increasing NMDA receptor-mediated nerve damage and glucose-induced inflammation and oxidative stress (138–140)] is one of the mechanisms by which insulin achieves its neuroprotection (141). In addition, insulin exerts its neuroprotective actions by blood glucose reduction and other mechanisms. These mechanisms include promoting glycogen synthesis, enhancing the functions of the neurotransmitters, and suppressing the process of neuronal apoptosis and necrosis, in addition to its antioxidant and anti-inflammatory effects (141, 142). Insulin is given through two different modes of administration, either standard or intensive modes. Standard insulin therapy involves subcutaneous insulin administration on a sliding scale every 6 h as needed to keep a blood glucose level between 80 and 179 mg/dL, while intensive insulin therapy is referred to as the continuous infusion of insulin intravenously as needed to keep the blood glucose levels between 80 and 130 mg/dL. Both methods are compared by the SHINE trial, a phase III RCT (143). SHINE examined the 90-day outcomes based on the modified Rankin scale score between the standard and the intensive insulin therapy. The trial concluded that there were no notable differences in the outcomes between the two groups (144), thereby concluding either method of administration can achieve the benefits of insulin therapy in stroke patients. Another route of insulin administration that has proved its efficacy on animals is intranasal insulin administration. Zhu et al. showed promising results from their experiment on animals, concluding that intranasal insulin could ameliorate neuronal dysfunction in mice with hemorrhagic stroke. Human studies are needed to check the efficacy of this method application, as it is recommended to use insulin therapy with any cases of AIS regardless of the glucose level (142).

#### Neu2000KWL

Neu2000KWL is a new multitarget drug designed to prevent excitotoxicity and oxygen toxicity by reducing NMDA receptor activity and the toxic effects of free radicals (145). Many ongoing trials are investigating the effectiveness of Neu2000KWL in protecting brain cells in cases of AIS. The ENIS I trial examines the safety and efficacy of Neu2000KWL in ischemic stroke (146). Once thromboembolism has occurred, nine consecutive infusions of Neu2000KWL at 12-h intervals are administered just before endovascular therapy with mechanical thrombectomy to follow (*NCT02831088)* (147). The trial is still ongoing and aims to investigate the safety of neuroprotection with Neu2000KWL and its effect on improving the outcomes when given before EVT. The outcomes of the trial were compared based on the modified Rankin scale at 3 months.

Another ongoing trial exploring the efficacy of Neu2000KWL as a neuroprotectant in patients with AIS is being processed in Beijing, China. The trial tests the outcomes of AIS after administration of Neu2000KWL infusion within 6 h from the onset of stroke symptoms followed by nine consecutive infusions at 12-h intervals. It will also compare the efficacy of different doses of the neuroprotective agent, either low, middle, or high. The outcomes of the study will be measured based on the National Institutes of Health Stroke Scale score of the involved patients. A score of 0 to 1 within 14 ± 2 days from the first injection represents a favorable outcome in those patients. Although the drug has not been approved yet by the US Food and Drug Administration to be used in cases of AIS, many reports see a promise in the efficacy of the drug as a neuroprotective medication.

#### Nimodipine

The calcium channel blocker nimodipine has also been studied for stroke patients due to its vasodilatory effects. Furthermore, nimodipine was shown to have the most potent vasodilation of all the calcium channel blockers, and this has shown utility in multiple cognitive treatments. However, the overall benefits were suboptimal despite the stroke size (148, 149). Interestingly, those who received their medication intravenously showed worse outcomes compared to those that received it by other means within a 12-h window (148, 149). Some of the benefits of nimodipine treatment include decreased hypertension-related intracranial vascular changes (150) and improved cognition (151–155). These benefits suggest nimodipine may play a neuroprotective role. When nimodipine was utilized early in subarachnoid hemorrhage patients, it decreased the neuronal damage induced by vasospasms (156). However, it has not been proven to reduce the incidence of angiographically documented delayed cerebral vasospasms (157–160).

#### Antioxidants and Antibodies

Oxidative stress is known to cause great insult to the brain in cases of ischemia. It is caused by oxygen and nutrient deprivation to brain cells, resulting in an imbalance between free radicals and the opposing antioxidant mechanisms (56, 161). Levels of antioxidants have been shown to correlate with the outcomes of stroke patients. Two naturally occurring and easily administered antioxidants that have been tested are uric acid and albumin. They have various mechanisms for why they work to reduce stroke risk, including removing harmful free oxygen radicals (162–164). The oxidative stress of free radicals is via the compounds superoxide, hydroxyl, nitric oxide, and peroxynitrite. The damage of these free radicals is further worsened by the reduction in scavenger enzymes in stroke due to energy depletion (165). Several antioxidant therapies are used in patients of AIS as neuroprotective agents to reduce the damage predisposed by the oxidative stress caused by increased free radical concentration (161). Edaravone is a free radical scavenger and a potent antioxidant against the peroxidation of the phosphatidylcholine liposomal membrane and reduction of ROS generation (166, 167). Many trials in Asian countries were first performed to check its safety and efficacy as a neuroprotectant (167). Its utilization as a neuroprotectant was first approved in Japan in 2001, and its administration in AIS is recommended by the Japanese guidelines in stroke management (168). In their systematic review of RCTs, Feng et al. (169) showed that the use of edaravone in patients with AIS resulted in substantial improvement in the neurologic functions as compared to placebo. Another recent large retrospective observational study suggested using edaravone in combination with endovascular reperfusion therapy to get the best outcomes of the intervention (170).

When brain ischemia occurs, ROS generation (edaravone inhibits this process) opens the mitochondrial permeability transition pore. Triggering of the mitochondrial permeability transition pore results in further release of ROS, a process called ROS-induced ROS release. This process is inhibited by cyclosporin A (167, 171). Cyclosporin A was shown to reduce the reperfusion-induced metabolic damage of brain nerve cells (172). Nighoghossian et al. demonstrated that IV cyclosporine A did not significantly reduce the infarct size when used in combination with thrombolytic therapy. However, it potently reduced the proximal cerebral artery occlusion resulting in successful recanalization (173). Due to the complementary role of both, edaravone and cyclosporine A can be used effectively in combination to achieve the best neuroprotection (167).

A multicentered trial on anti-CD49d (integrin alpha 4) antibodies has been completed to assess their ability to stop the migration of inflammatory cells (i.e., leukocytes) into the brain after the onset of ischemic stroke. This treatment would lower the generation of free radical damage mediated by the infiltrating peripheral leukocytes during the inflammatory response (174). Sadly, initial findings showed that only permanent stroke victims benefitted from the anti-CD49d treatment, whereas transient ischemic stroke patients did not (175). Recently, in an attempt to stop leukocyte adhesion and migration during stroke infarct, natalizumab (a monoclonal antibody against the leukocyte adhesion molecule α4 integrin) was tested in preclinical trials (175–177). The safety and efficacy of natalizumab on human participants were evaluated in the RCT ACTION. This trial showed no improvement in infarct size when administration occurred within a 9-h window of ischemia onset (178). However, there were improvements in the functional outcomes following its use, enhancing its role in neuroprotection (178).

#### Positive Modulators of GABA Receptors, Transaminase Inhibitors, or Transporter Blockers

The rapid and transient GABA elevation in the extracellular space during cerebral ischemia acts like most neurotransmitters (179, 180). Early in the reperfusion phase, disruption of GABA-mediated neurotransmission can lead to ongoing neuronal excitability and probably neuronal death (181, 182). Importantly, the different receptors of GABA have been shown to affect the brain differently, and utilizing the different traits of the glutamate and GABA receptors has helped reduce toxicity and increase efficiency (52).

There is a transient increase in ligands binding to inhibitory GABA-A receptors in the preconditioned *in vivo* hippocampus (183). Neuroprotection is abolished with weak antagonism of the GABA-A receptors and bicuculline as determined by viewing ischemic preconditioned rat hippocampal slices (184). Activation of GABA-B receptors has been suggested to support the role of hippocampal neuron neuroprotection (84). Simply put GABA-A receptors are associated with ischemic intolerance, whereas GABA-B receptors seem to be a component of neuroprotection (84, 183).

When endogenous GABA levels are pharmacologically increased, both GABA-A and GABA-B receptors are triggered, contributing to neuroprotection (185). Thus, GABA drugs that function as agonists or positive modulators of GABA receptors, GABA transaminase inhibitors, or GABA transporter blockers provide neuroprotection in the preclinical environment (186). When utilizing GABA in treating ischemic stroke, one will have to consider where the stroke occurred. The expression of both GABA-A and GABA-B receptors decreases in different brain regions of rodents subjected to transient ischemia (187).

#### Glial Cells as a Neuroprotection Target

**Figure 2:**
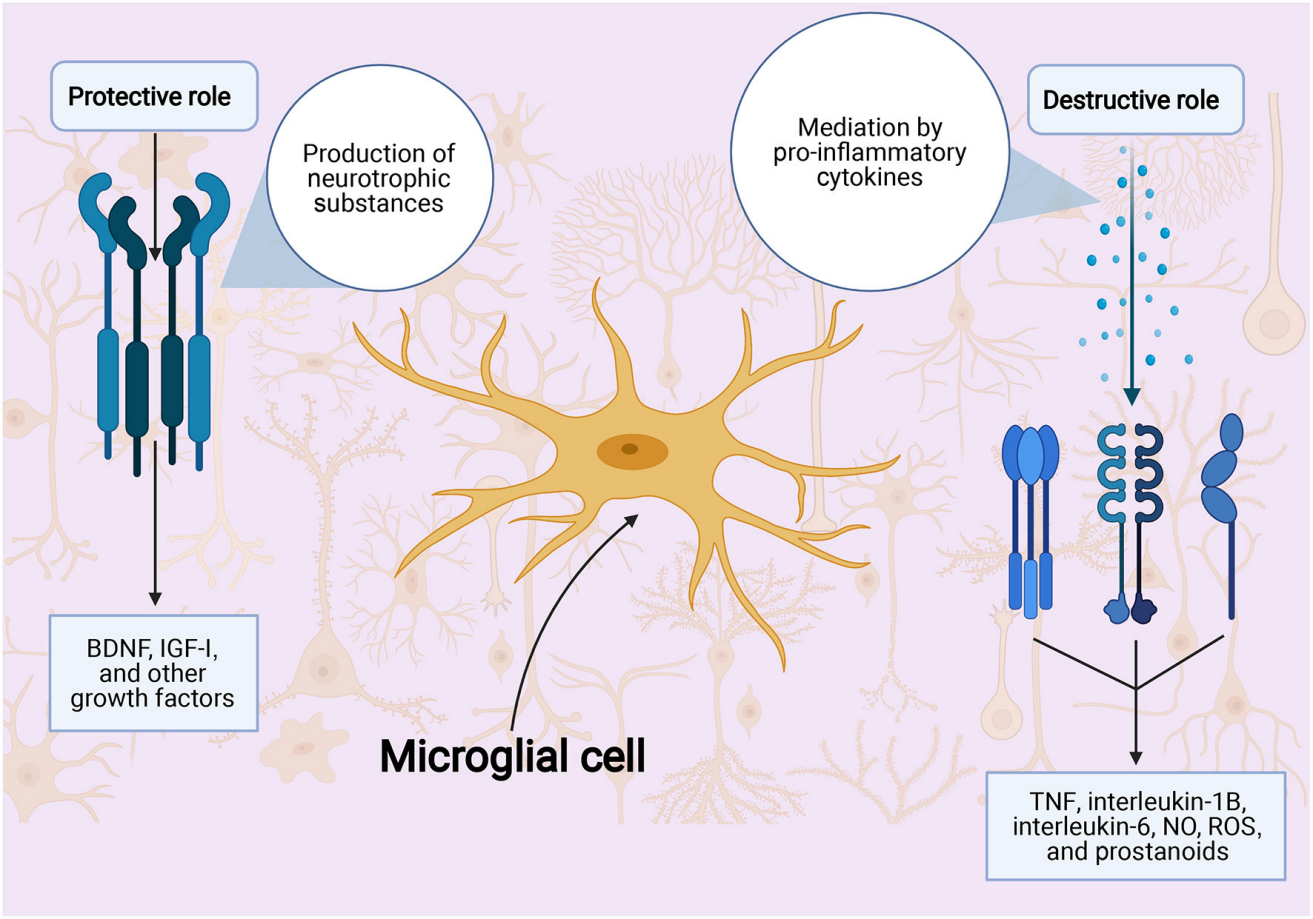
Role of microglial cells in ischemic stroke injury. BDNF indicates: NO, nitric oxide; ROS, reactive oxygen species; TNFα, tumor necrosis factor α.

The cells of the brain have been shown to play a role in the ischemic stroke response. Cells that have been shown to play a role include the microglia and the astrocytes. While they both release proinflammatory cytokines in ischemic stroke, the microglia also release toxic metabolites and enzymes (188, 189), whereas the astrocytes release neuroprotective factors (190). In addition, microglial cells and monocytes also play a role in ischemic stroke injury via increases in free radicals through elevated nitric oxide synthesis (22) (Figure 2).

The sphenopalatine ganglion is known to have multiple benefits when stimulated, including increased collateral blood flow to the brain and reduction in the size of infarct in ischemic stroke patients. A recent study found that it is safe to stimulate the sphenopalatine ganglion within the first 8 to 24 h of stroke symptoms. Although these are still only clinical trials, they show promise in benefit outcomes of stroke, but not in mortality (191). It has also been reported that upon sphenopalatine ganglion stimulation *in vitro*, there was normalized brain function with decreased blood-brain barrier dysfunction when applied 24 h after ischemic stroke (192). Changes caused by increased inflammatory markers can lead to changes in the endothelial cell layer, causing blood-brain barrier dysfunction (188) and more wound healing post-recovery due to infiltration of leukocytes from circulation (193–196). These findings suggest that cellular regulation and stimulation may be helpful in ischemic stroke patients even when outside the therapeutic window.

#### Stem Cells as a Neuroprotection Agent

The basis of using stem cells in the therapy of ischemic stroke patients is to replace the damaged neuronal tissue with new healthy cells. In recent years, experiments have proven stem cells to be effective in treating AIS while having very few adverse effects (197). The mechanism behind this includes anti-inflammation, antiapoptosis, antioxidative, blood-brain barrier protection, and the promotion of angiogenesis and neurogenesis (198, 199). In addition, stem cells have been shown to lend support to the at-risk tissues surrounding the infarct itself (200, 201). Thus, due to its relatively low cost and multiple delivery routes, the use of stem cell therapy in stroke patients should be investigated and utilized further.

## Future Implications

We believe that our paper has adequately discussed the potential therapeutic and neuroprotective modalities for improving neurologic outcomes after AIS. We also believe that the currently ongoing trials about the various neuroprotective agents will add to the literature and lead to better evidence by indicating or inhibiting the previous and currently announced potential neuroprotective modalities. Future trials investigating the clinical outcomes of AIS patients using neuroprotection strategies/agents are encouraged. It is also expected that these trials will overcome the current limitations, regarding heterogeneity in reporting a standardized measure of functional and clinical outcomes of patients. Furthermore, future investigations should attempt to validate proven efficacious *in vitro* neuroprotective agents to make the best use of them.

Future research should also focus on the efficacy of adding neuroprotective treatment to endovascular treatment, as suggested that reperfusion together with neuroprotection is associated with enhanced outcomes after AIS. However, rapid reperfusion might be limited by inducing reperfusion injury. This has been attributed to increased ROS levels in previously ischemic tissues, secondary to reperfusion-related increases in oxygen levels. Therefore, it can be suggested that ROS-related neuroprotection modalities might be the ideal candidates to combine with reperfusion therapy and should be an area of focus for future investigations.

Moreover, stem cell-based treatment modalities are also promising treatments that will potentially influence the prevention of AIS and neuroprotection. Previous investigations conducted in preclinical models of AIS have been published, showing promising results (202, 203). Accordingly, many intentions have been declared to conduct similar human trials for adequate validation of these modalities, although the early findings are not appreciably promising (204).

Future preclinical investigations should also aim at enhancing the mechanism of action and targeting of neuroprotective agents. According to the Stroke Treatment Academic Industry Roundtable (STAIR) X, the term neurovascular unit (NVU) can be used to identify different brain parts that are at higher risk of ischemia. It has been shown that NVU includes various cell types, like endothelial cells, astrocytes, and neurons that are susceptible to ischemia (205). Accordingly, future research studies might concentrate on neuroprotective agents enhancing the prognosis of different NVU areas (206).

## Conclusion

The management of AIS is time-dependent and needs urgent implementation to enhance prognosis. In the present literature review, we have discussed the advantages and complications of the most commonly described therapeutic modalities, in addition to the currently available neuroprotective agents and the roles they play in enhancing prognosis. Urgent approaches that can help stabilize the state of the patient can enhance the outcomes. Validating the results of the ongoing trials and the current *in vitro*-proven efficacious modalities is needed.

## Author Contributions

SG, WB, RK, and DK contributed to the conception, design of the work, and revising it critically for important intellectual content. AR handled the reviewer comments and revised the manuscript accordingly. All authors contributed to the acquisition, interpretation of data, drafting the work, agreed to be accountable for all aspects of the work in ensuring that questions related to the accuracy or integrity of any part of the work are appropriately investigated, resolved, and approved the final version of this manuscript to be published.

## Conflict of Interest

The authors declare that the research was conducted in the absence of any commercial or financial relationships that could be construed as a potential conflict of interest.

## Publisher's Note

All claims expressed in this article are solely those of the authors and do not necessarily represent those of their affiliated organizations, or those of the publisher, the editors and the reviewers. Any product that may be evaluated in this article, or claim that may be made by its manufacturer, is not guaranteed or endorsed by the publisher.
